# The Evolving Role of Laboratory Doctors: A Shift towards Patient Consultation

**DOI:** 10.31729/jnma.8838

**Published:** 2024-12-31

**Authors:** Vivek Pant

**Affiliations:** 1 Department of Clinical Biochemistry, Samyak Diagnostic Pvt Ltd, Jawalakhel, Lalitpur, Nepal

**Keywords:** *consultation*, *counselling*, *doctors*, *laboratory*

## Abstract

In developing countries such as Nepal, the scarcity of doctors and their constrained consultation time often hinder comprehensive patient education regarding laboratory pre-test preparations and post-test results counselling. This study examines the potential of integrating laboratory doctors and direct patient consultations as alternatives to improve patient management. By piloting this practice at our institution, this viewpoint highlight that the laboratory doctors, with their specialized knowledge, can effectively communicate pre-test and post-test information, thereby enhancing patient understanding and compliance. This article also discusses the practical implications of this model and offer recommendations for policy adjustments to facilitate this integration

## INTRODUCTION

Historically, the role of laboratory doctors in Nepal, as in many other countries, was largely behind the scenes. Their primary responsibilities included analyzing specimens, ensuring the quality control of laboratory processes and interpreting laboratory data for the benefit of treating physicians. Patient interaction was minimal, with clinicians and other healthcare providers acting as intermediaries between patients and laboratory doctors. Clinicians were solely serving as a source of interpretation of the laboratory information however patients may learn about the meaning of laboratory results through independent sources such as internet Recently, a transformative change has emerged in the way laboratory doctor's practice.

## TRANSFORMATION IN PRACTICE OF LABORATORY DOCTORS

Shift in laboratory doctor's practice has been driven by several factors including increased patient awareness, technological advancements and growing emphasis on comprehensive care, which includes pre-test and posttest consultations to better manage health conditions. Apart from these apparent factors, the growing shift of laboratory professionals towards direct patient consultation is primarily driven by the scarcity of clinicians and the limited time they can dedicate to pretest and post-test consultations. Laboratory doctors, with their expertise offer more precise explanations, enhancing patient understanding and ensuring better preparation and follow-up.

As laboratorians, direct interactions with patient help us to keep ourselves updated. We need patients to inform our findings, to clear laboratory related doubts among patients, to reconnect with the need for new tests and to encourage our fraternity. My interactions with patients have also allowed me to learn about what patients are required to know about laboratory investigation. Reading descriptions in a medical text is a different matter from listening to patients talk about how difficult is it to execute some pre-test preparation. The benefits of my interactions with patients sometime go beyond the result interpretation. I had a chance to diagnose a case of pseudo-hypertriglyceridemia in patient who was prescribed a bucket of medicine for last 13 years without significant outcome.^1^ My personal conversations with patients have also reinforced the tremendous need for my research. When the news of my findings were disseminated in scientific journals and local health magazine, the volume of calls and emails I received from patients led me to suspect that the incidence of patient requiring direct consultation from laboratory doctors might be much higher.

## POTENTIAL AREAS OF COUNSELLING BY LABORATORY DOCTORS

When I searched the medical literature for references to direct patient consultation, I found very few literatures.^2,3^ Laboratory results are often complicated and its interpretation requires knowledge of normal patterns, laboratory statistics and biological variation. To minimize uncertainty, patients can be counselled by laboratory doctors before tests are ordered and cautioned about variable information obtained via internet Counselling by laboratory doctors could be continued when results are available. My conversations with patients have been enormously reassuring because it has allowed me to better understand patients' medical histories and symptoms, leading to more informed interpretations of test results. I also noted improved job satisfaction from direct patient interactions.

**Table 1:** Critical areas where pre-test consultation can significantly enhance patient care and diagnostic accuracy.

Area of Pretest Consultation	Examples	Benefits
Selection of Appropriate Tests	Avoiding thyroid test during other illness Recommending fructosamine test instead of HbAlc in anemic conditions High vitamin B12 level could be seen if patient is taking over the counter multivitamin supplements Altered liver enzymes could be seen if patient is taking Ayurvedic medicine	Ensures accurate diagnosis, avoids unnecessary testing and reduces patient anxiety and costs
Understanding Test Preparation	Expect low or high blood tacrolimus test while taking drugs such as Rifampicin, Clarithromycin or having diarrhea	Ensures patient compliance with preparation protocols
Interpreting Test Limitations	Immunochromatography based test have high rate of false positive and false negative results with numerous potential interferences	Helps set realistic expectations based on test results Helps to understand diagnostic performance of screening tests
Clarifying Test Procedures	One can vomit during oral glucose tolerance test leading to false glucose reading Explaining correct sequence for dynamic endocrinological tests	Reduces patient anxiety and improves cooperation during the test
Understanding Results Timing	Genome sequencing and karyotyping takes certain time and there are special tests that a laboratory runs once or twice a week only based on their test volume or other uncertainties	Manages patient expectations and aids in timely follow-up and treatment planning
Risk Assessment	Choosing correct marker based on patient's history and symptoms for hepatitis B virus or HIV Discuss difference between TB QuantiFERON and Mantoux test Explaining risk associated with radiolabeled test such as urea breath test especially in children and pregnancy Explaining sensitive tests such as related to genetics, cancer, pregnancy, sexually transmitted diseases, or mental health	Enhances patient safety by identifying and mitigating risks associated with testing
Follow-Up Tests and Referrals	Add bile salt test as a reflex test if liver enzymes are high in late pregnancy Advise for hemoglobin electrophoresis if variant window detected in HPLC chromatogram when running HbAlc Manual counting for platelets if dengue serology is positive	Ensures comprehensive patient care and thorough investigation of health concerns

**Table 2:** Critical areas where post test consultation can significantly enhance patient care.

Areas of Post-test Consultation	Description/Examples	Benefits
Interpretation of Complex Results	Understanding genetic mutations, their implications, and advising on preventative measures or treatments	Enhanced comprehension of genetic risks, personalized preventive strategies, and tailored treatment plans
	Clarifying hormonal assays, hormonal imbalances and their impact on health	Improved management of endocrine disorders
Discussing Variations in Results	Explaining origin of variability such as differences in equipment, methodologies, and protocols across laboratories, as well as from pre-analytical and biological factors	Clear guidance on follow-up testing
Clarification of Ambiguous Findings	**Unexpected Findings** Addressing unexpected results that were not anticipated based on clinical presentation	Timely identification of potential issues, appropriate adjustments to diagnostic or treatment strategies and avoidance of detrimental self-interpretation of test results
	**Borderline/Equivocal Results** Providing context and next steps for results that are not clearly positive or negative Reduced patient anxiety and confusion, clear guidance on follow-up testing or treatment
Personalized Treatment Plans	Pharmacogenomics: Advising on drug metabolism and potential adverse effects based on genetic profiles	Enhanced drug efficacy, reduced adverse effects, and personalized medication regimens
Risk Assessment and Prevention	**Cardiovascular Risk** Interpreting tests related to heart health and recommending preventative strategies	Informed lifestyle changes
	**Infectious Diseases** Explaining serological and molecular test results for infections and advising on treatment options	Accurate diagnosis of disease course, effective treatment plans, and prevention of disease transmission

## CHALLENGES AND FUTURE DIRECTION

Implementing this direct consultation model requires overcoming several challenges, including training laboratory doctors in patient communication, restructuring workflow to allow time for consultations, and ensuring that such interactions are integrated into the broader healthcare system. Additionally, there must be careful consideration of the scope of practice to avoid conflicts with primary care physicians and specialists.

To sustain and enhance this practice, several steps can be taken such as offering continuous training and development opportunities for laboratory doctors, providing necessary resources and infrastructure to facilitate effective consultations and promoting research and feedback to understand the impact of these consultations and to continuously improve the process.

Instead of attributing discrepancies in lab reports to errors or negligence, clinicians should also engage with laboratory doctors to gain insights into this model of patient counselling. By fostering a collaborative relationship, clinicians and laboratory doctors can work together to ensure accurate diagnoses and effective patient management, ultimately enhancing the quality of healthcare and reducing unnecessary repeat testing and associated costs.

## CONCLUSIONS

The evolving role of laboratory doctors in encompassing direct patient consultations marks a significant advancement in the healthcare system. As healthcare systems worldwide strive for more patient-centered care, incorporating direct consultations by laboratory doctors could play a crucial role in achieving these goals by engaging with patients before and after tests.
